# Using alternative teaching and learning approaches to deliver clinical microbiology during the COVID-19 pandemic

**DOI:** 10.1093/femsle/fnab103

**Published:** 2021-08-19

**Authors:** Lovleen Tina Joshi

**Affiliations:** School of Biomedical Sciences, University of Plymouth, Drake Circus, Plymouth PL4 8AA, UK

**Keywords:** clinical microbiology, teaching, engagement, online, in-person

## Abstract

The COVID-19 (coronavirus disease 2019) pandemic has had significant impacts upon higher education teaching. Clinical microbiology teaching is primarily focused on a combination of practical skill development and didactic delivery of content. In the pandemic, the absence of in-person teaching has led to educators adapting in-person content for online platforms and delivery. This commentary covers alternative innovative and engaging teaching approaches to deliver clinical microbiology content during the COVID-19 pandemic.

## Teaser

Online teaching of clinical microbiology during COVID-19 pandemic to engage students.

## INTRODUCTION

Severe acute respiratory syndrome coronavirus 2 (SARS-CoV-2), causative agent of coronavirus disease 2019 (COVID-19), has had unprecedented global health, economic and societal impacts (Mou 2020; Wu *et al*. 2020). SARS-CoV-2 is a respiratory viral pathogen able to readily transmit from infected individuals via droplet or airborne transmission (Morawska and Milton 2020; Prather *et al*. 2020). As SARS-CoV-2 spread uncontrollably across the globe, pressure across healthcare services grew as hospital admissions and mortality rates increased (Lee *et al*. 2020). On 11 March 2020, the World Health Organization declared COVID-19 as a pandemic and on 23 March 2020 the UK Government declared a national ‘lockdown’ where ‘stay-at-home’ measures, including social distancing, were implemented to protect UK citizens and preserve National Health Service healthcare capacity (Lee and Morling 2020).

The consequences of ‘stay-at-home’ lockdown measures were unprecedented for higher education (HE) teaching. Prior to the pandemic, some HE institutions had provisions for instant accessible learning via the use of digital lecture content capture platforms to enhance the student experience (Biggs and Tang 2011; Newton *et al*. 2014). There are arguments that this has been primarily driven, in the United Kingdom, by increased student expectations in the face of paying tuition fees, i.e. where students are perceived as the ‘consumer’ and HE institutes as ‘service providers’ (Myers 2013; Wong and Chiu 2019). However, when the pandemic hit, online transition was forced upon HE institutions causing a complete shift from in-person delivery of education to online teaching (Lemay, Doleck and Bazelais 2021). In such difficult circumstances, how is it possible for lecturers to successfully engage and motivate students?

## ADAPTING TO ONLINE TEACHING

Clinical microbiology is the diagnostic study of infectious microorganisms and their role in human disease (Reller *et al*. 2001). Effective teaching and learning of clinical microbiology relies on a combination of ‘hands-on’ practical active learning techniques and didactic delivery of essential scientific information, with the latter being encouraged due to its perceived ‘economical and efficient’ delivery to large student cohorts (Rutherford 2015; Stevens *et al*. 2017). Didactic lectures tend to use a combination of behaviourist and constructivist learning approaches resulting in passive student learning (Keough and Naylor 1996). Thus, motivating students to engage with STEM (science, technology, engineering and mathematics) subjects via didactic delivery in a teacher-centred approach is not necessarily conducive to successful student learning. To mitigate potential lack of student engagement, HE lecturers often combine traditional teaching techniques with small group teaching, flipped classroom techniques, gamification and quizzes (Ashwin *et al*. 2015).

Face-to-face teaching also allows educators to tailor to individual needs in real time and answer queries directly for the student in a student-centred approach, especially in a practical laboratory context (Tofade, Elsner and Haines 2013). In the case of teaching, employing a constructivist approach via active learning can enable students to teach each other through understanding and building upon frameworks of microbiological knowledge (Piaget 1970; Hunt and Chalmers 2013). During the pandemic, the transition from teaching face-to-face to online posed a challenge when attempting to engage students and encourage active learning (Table 1).

**Table 1.** Pros and cons of online and in-person teaching delivery.

Teaching delivery	Pros	Cons	References
In-person (face-to-face)	Social contact; feeling of belonging in learning community; direct communication	No anonymity when students ask questions, unless interactive digital technology platforms are used (Mentimeter, etc.)	Redmond (2011); Ashwin *et al*. (2015); Rutherford (2015)
	Easy to assess student knowledge in the form of questions, etc., more so in practical sessions	Students can be distracted by their peers during large cohort sessions and miss essential content	Brockman *et al*. (2020); Alvarez (2021)
	Face-to-face feedback can be delivered immediately	Teaching can become too formalized in an attempt to manage large cohorts	Biggs and Tang (2011)
	Easier for group work		Efthimiou and Tucker (2021)
	The educator can explain concepts using a whiteboard in real time		
	More options for use of blended learning techniques		Sancho *et al*. (2006)
Online	Flexible for students who are unable to attend sessions due to family or other commitments (inclusive)	Students may require feedback and clarification of difficult concepts outside of the session—usually via email or extra Zoom sessions	Thomas (2010); Newton *et al*. (2014); Lemay *et al*. (2021)
	Assessments, including examinations, are often timed and ‘open book’ allowing for flexibility	Difficult to assess student knowledge online in sessions throughout course delivery; students can ‘cheat’ or collude during online examinations	Bilen and Matros (2021)
	Virtual classrooms mean the guest speakers can be invited to deliver sessions online from anywhere in the world	Being in long didactic sessions can result in tired students who have ‘checked out’	Dumford and Miller (2018); Jiang (2021)
	Instant access to module content and lecture recordings	Students can still be distracted with their microphone muted and their cameras turned off	Euzent *et al*. (2011)
	Breakout rooms, screen sharing and virtual whiteboards can be used to create variety and stop distractions	Online teaching, without social contact, can cause a significant mental health toll on students	Rutherford (2015)
	Informal teaching techniques can be used to gain interesting feedback and comments from students in a ‘safe’ anonymous environment	Academic preparation time doubles for each online session; academic educator ‘burnout’	Tofade *et al*. (2013); Wong and Chiu (2019); Jiang (2021)

During a pandemic, it is likely easier for microbiology concepts to be put into immediate and relevant context. An example of this is in teaching epidemiology, for example outlining John Snow's use of the scientific method in investigating the 1854 cholera outbreak in Soho, and relating this back to the current SARS-CoV-2 epidemiological investigations (Caplan, Kennedy and Neudecker 2020). Impacts of asymptomatic transmission, especially in the current context of SARS-CoV-2, can be explained by using the example of ‘Typhoid Mary’ as an asymptomatic transmitter of *Salmonella typhi* (Brooks 1996; Marineli *et al*. 2013). Employing case study and infection scenarios online is also possible through use of collaborative learning, where students can be put into breakout rooms to examine the scenarios and provide feedback to the cohort (Rutherford 2015).

Another way of making online content more interesting for clinical microbiology students is to relate the content to popular culture via investigative case studies (Tomes 2002). An example of this is adapting scenarios from the reality television programme Love Island to hypothetically map transmission of sexually transmitted infections (STIs) and explain symptoms among the contestants. Love Island is a reality-based television dating programme where ‘single’ contestants spend two months in a villa in Spain to find a partner. On arrival, contestants are asked to pair up with a partner, i.e. ‘coupling up’, and anyone left ‘single’ has to leave the programme (L'Hoiry 2019). The contestants take part in challenges in their ‘couples’, kiss and can choose to become more intimate in the ‘Hideaway’. The infection case-study scenario is adapted from this where fictional contestants can ‘couple up’ and be given a hypothetical STI (or not). Students can in groups, using a trail of informative symptomatic clues given in a document via breakout rooms, figure out who originally had the ‘STI’ from the love connections made. The final assessment of student understanding is to explain the results of a PCR (polymerase chain reaction) test to determine which antibiotic-resistant ‘superbug’ the contestant had (one such scenario used *Neisseria gonorrhoeae* as the STI). This adapted learning scenario was successfully tried in practice as (i) students enjoyed the investigative nature of the learning and (ii) the programme is already popular with Generation Z students. Generation Z students are defined as being born post-1995, have yet to enter the workforce and are digitally savvy, highly connected and make fast decisions (Cilliers 2017; Dimock 2019).

Gamification, where game techniques are applied in a nongame environments, is being increasingly used within HE as an attractive substitute for didactic learning (Plass, Homer and Kinzer 2015; Efthimiou and Tucker 2021). Gamification allows students to engage with ‘drier’ teaching content and is thought to increase student retention of learning material (Robinson, Turner and Sweet 2018). While it is easy to undertake gamification activities using physical board games in small group teaching scenarios, it is also possible online. One such way is by playing games such as ‘STI Bingo’ online with students over Zoom, where symptoms of STIs can be called out by the educator as per the game's instructions, and when the student has crossed off a full set of symptoms on their card, they can shout out what STI they potentially have (BPAS 2021). Another way of employing gamification online is by use of applications (apps) on mobile phones or computers that are cheaper alternatives to physical materials when teaching large cohorts. Examples of this include ‘Outbreak’ and the Plague Inc. games app (Ndemic Creations, UK) that can be played on various platforms such as mobile phones and can engage students with learning about the effects of pathogens with specified traits on a population (Robinson, Turner and Sweet 2018; de Almeida, Taschner and Lellis-Santos 2021).

Tapping into academic networks to find guest lecturers on a relevant topic is also a good way of increasing student engagement. High-profile speakers who have been involved in the pandemic can be asked to deliver real-life information to students that increases their interaction, enthusiasm and understanding of the relevance of ‘drier’ taught content (Fahnert 2016). One such example was asking a contact who specialized in COVID-19 research within Public Health England to deliver a guest lecture online. The students were inspired by this lecture that covered the most recent developments in the pandemic. The guest lecturer was secured in advance of the lecture due to being in high demand. One of the key benefits of having guest lectures online is the reduced need for travel, more efficient use of time and the fact that these lectures remain recorded for students to refer to anytime.

Of course, other methods to engage students online include using props, such as GIANTmicrobes, Modbury, Devon, UK, to show students pathogenic characteristics of microorganisms in a crude but safe and fun format (Jermy 2016). GIANTmicrobes are plush toys of microbes that can be used as gifts or educational aids for adults and children. They come in a range of microorganisms from bacteria such as *Vibrio cholerae* to viruses such as Ebola and SARS-CoV-2. GIANTmicrobes are a highly effective way of teaching some basic clinical microbiology to students without the use of a laboratory. For example, when teaching students online about a certain microorganism, such as SARS-CoV-2, the GIANTmicrobes plush toy can be shown to students online to demonstrate its key features such as ‘spikes’. The same can be said for the use of real-time sequencers such as the Oxford Nanopore MinION sequencer that can be safely and successfully used to demonstrate DNA sequencing in real time online or in person (Salazar *et al*. 2020).

Practical laboratories in clinical microbiology are essential learning environments for students to obtain hands-on practical skills and develop professionally. This experiential learning is not possible through didactic lectures; however, during the pandemic alternatives needed to be sought in the absence of in-person clinical microbiology teaching. The skill sets required include safe working practices, and the ability to utilize aseptic techniques and handle microorganisms (Noel *et al*. 2020). Attempts to substitute in-person learning include use of videos to demonstrate key techniques within the laboratory, where the educator is filmed demonstrating tailored microbiological techniques, such as streaking an agar plate. While this is no substitute for hands-on learning, students can be encouraged to safely practise some techniques at home using everyday items. For example, the streak techniques can be practised at home using jelly set in a bowl and a piece of blunt plastic cutlery to streak chocolate sauce in the usual streaking format, the idea being that students can ensure the jelly is not broken when streaking (Madigan, Martinko and Parker 2017). In the case of the educator not being able to physically record the techniques, the *Journal of Visualized Experiments* (JoVE) has a repository of videos, but does require a subscription. Of course, not all techniques can be practised in this way, and hence there is a potential role for use of virtual online laboratories in the pandemic. An example of this is Labster that provides laboratory simulations at a subscription cost (Alvarez 2021). However, considering the core traditional microbiology skill set required by future microbiologists, online learning is a poor substitute for in-person learning where immediate, tailored feedback can be given to students.

In the author's case, it was possible to deliver microbiology practicals during the semester by provisioning extra practical sessions that allowed us to stay safe from COVID-19 and adhere to government social distancing guidelines. For those unable to deliver during semester, planned summer laboratory ‘catch-up’ classes are an excellent way of addressing the lack of in-person laboratory learning. One concern, however, is how many students do attend these additional classes over the summer period.

## SUMMARY

It is likely that online teaching will continue in some format while the COVID-19 pandemic continues. An ideal scenario would be blended learning where successful elements of online teaching are combined with in-person teaching to deliver an appealing student experience (Sancho *et al*. 2006). As a microbiology educator, I did try many of the above techniques to improve student engagement and information retention. My lectures were didactic but used GIANTmicrobes to demonstrate key microbiological features, and securing a guest lecturer from Public Health England microbiology enhanced the new SARS-CoV-2 content I had incorporated into the module. I delivered workshops via breakout rooms where students could collaboratively work on infection case-study scenarios, such as STIs and general clinical cases. The feedback from these alternative approaches was overwhelmingly positive. Moreover, covering the background of epidemiology starting from John Snow and the scientific method to current epidemiological methods to investigate outbreaks improved the students’ understanding of the current pandemic. In fact, the epidemiological steps in outbreak investigations formed part of the students’ examination assessment in June 2021. I also employed gamification by playing STI Bingo with the students online, which consolidated their understanding of clinical symptoms of STIs. These sessions not only are engaging for the students but can also be great fun for the lecturer too. Indeed, a future clinical microbiology course is likely to be blended in format, combining online platforms for guest lectures, gamification, online quizzes and face-to-face didactic and practical sessions to enhance microbiology learning. This will require constant modification and trialling of alternative approaches to see which works best across cohorts.

**Figure 1.**
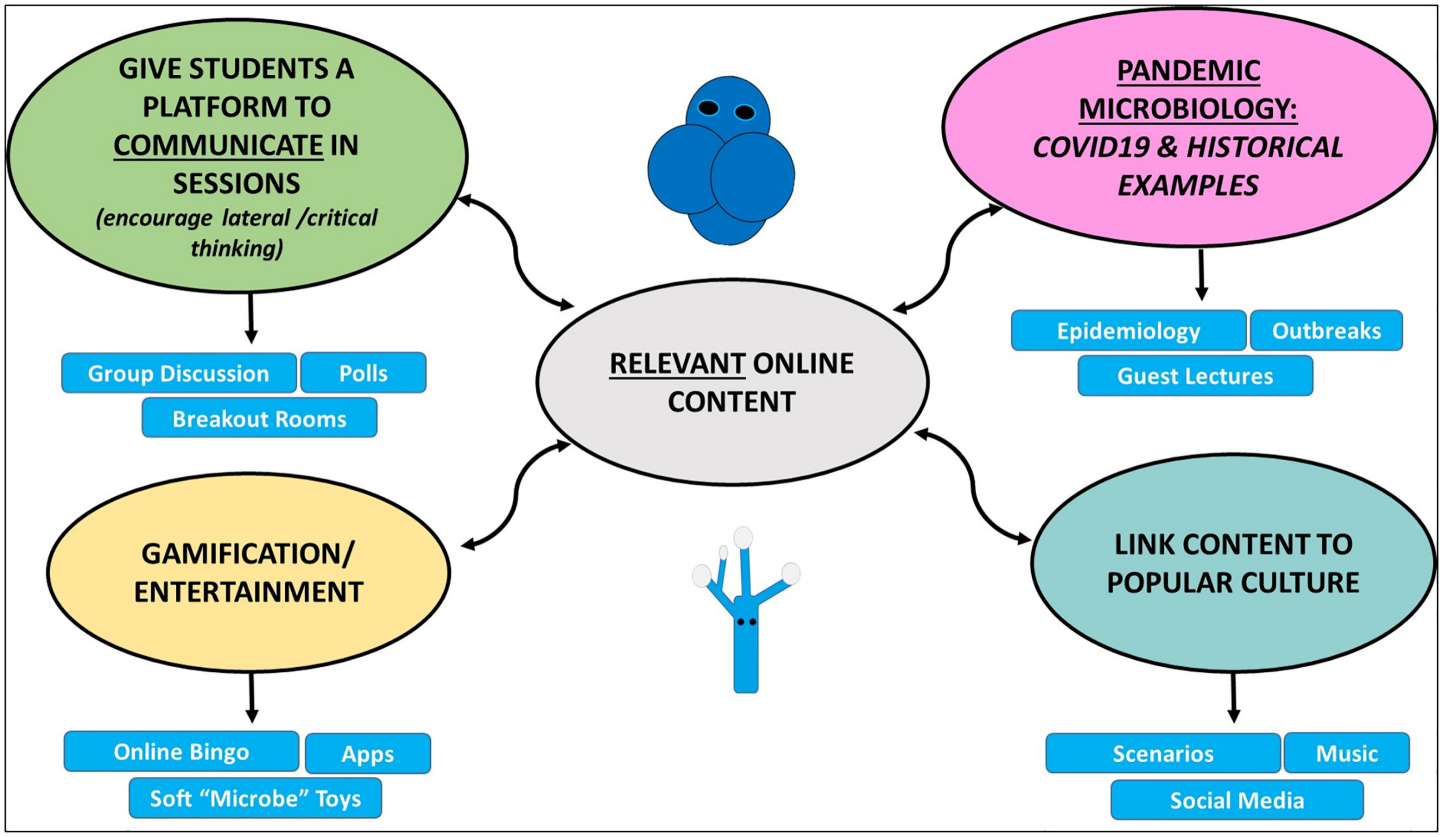
General summary of relevant content suitable for online teaching delivery. Incorporating popular culture, gamification and current events can contribute to a more engaging learning experience for students online. This is applicable during a pandemic, or the approaches can be blended with in-person teaching.

Allowing students to communicate and provide feedback within online sessions is key to increasing engagement and a sense of being part of the learning community (Fig. 1). This is not true, however, for clinical microbiology laboratory skills that do require in-person teaching. This is essential to train the microbiologists of the future to safely handle clinical pathogens without compromising their professional development. Therefore, delivering extra practicals during semester was the best way we ensured that the student experience would not be compromised and that they would acquire essential skills required for the course—especially if accredited. The COVID-19 pandemic appears to have encouraged a renewed interest in the study of clinical microbiology. I have experienced an increase in students (84 in 2020) choosing to undertake the clinical microbiology module at final year compared with previous years (50 in 2019). From reading student feedback, the main driver for this increase is a desire to learn more about antimicrobial resistance, COVID-19 disease and microbiology. Engaging and innovative teaching has a significant and important role to play in providing microbiologists with the skills to tackle healthcare challenges, especially with the advent of the COVID-19 pandemic and the silent pandemic of antimicrobial resistance.

## ACKNOWLEDGEMENTS

The author wishes to express her sincere thanks to the students she has taught to date, and a special thanks to current students who have achieved their degree qualifications despite the turbulence of the COVID-19 pandemic. No funding was used to produce this commentary.
